# Dynamics of surface evolution in semiconductor thin films grown from a chemical bath

**DOI:** 10.1038/srep33136

**Published:** 2016-09-12

**Authors:** Indu Gupta, Bhaskar Chandra Mohanty

**Affiliations:** 1 School of Physics and Material Sciences, Thapar University, Patiala–147004, India

## Abstract

Dynamics of surface evolution in CdS thin films grown by chemical bath deposition technique has been studied from time sequence of atomic force micrographs. Detailed scaling analysis of surface fluctuation in real and Fourier space yielded characteristic exponents *α*_*loc*_ = 0.78 ± 0.07, *α* = 2.20 ± 0.08, *α*_*s*_ = 1.49 ± 0.22, *β* = 0.86 ± 0.05 and *β*_*loc*_ = 0.43 ± 0.10, which are very different from those predicted by the local growth models and are not related to any known universality classes. The observed anomalous scaling pattern, characterized by power law scaling dependence of interface width on deposition time differently at local and global scale, with rapid roughening of the growth front has been discussed to arise as a consequence of a nonlocal effect in the form of diffusional instability.

Recent developments in scaling invariance and universality have led to a growing interest in kinetic roughening theory with special attention being paid to studies on roughness evolution in thin films grown under far-from-equilibrium conditions^1^,^2^,^3^,^4^. The study of evolving surfaces provides insight to the fundamental growth dynamics and enables one to control the roughness of the films. Such a study is of high technological relevance in that roughness of thin films in multilayer structures affects electrical, optical, mechanical and catalytic properties, and hence, determines the eventual performance of devices^5^,^6^,^7^.

Typically roughness of a surface evolves as a consequence of simultaneous atomic scale processes such as direct addition of atoms on the growing surface from the surrounding, removal of atoms from the surface and motion of atoms along the surface or diffusive mass transport due to an existing or increasing chemical potential gradient^8^. The surfaces in many non-equilibrium growth models such as random surface recrystallization in the Eden model or ballistic aggregation are self-affining fractal which are described by the Kardar–Parisi–Zhang (KPZ) equation^9^
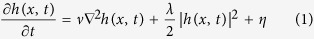
where *ν* accounts for the surface tension, *λ* is an “excess velocity” in the growth, and *η* is white noise. The self-affine patterns that the film surfaces develop into can be analyzed by the scaling properties of the surface fluctuations^10^. A number of applications of the KPZ equation were suggested based on the comparison of scaling exponents of surface roughness^1^.

The self-affine roughness is widely characterized by engaging it to a dynamic scaling form wherein the root mean square of the fluctuations of the surface height i.e. the interface width *w* defined as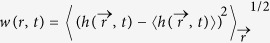, where *h* is the surface height and 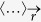 is spatial averaging in a system of size *L* and *r* ≤ *L*, evolves following a simple dynamic scaling known as Family-Vicsek relation:
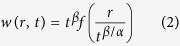
where the scaling function behaves as *f*(u) = constant and u^α^ for u ≫ 1 and u ≪1, respectively, *α* and *β* are the roughness and growth exponents respectively. The set of exponents corresponds to a specific universality class and is suggestive of the underlying mechanism that governs the evolution of roughness. Equation (2) suggests that for small *r* (i.e. *r* ≪ *t*^*β/α*^), *w* is independent of deposition time *t* and scales as *r*^*α*^, and independent of *r* for large *r* when it scales as *t*^*β*^. The crossover between these two behaviors occurs at *r = ξ*, the lateral correlation length, which signifies the distance at which the surface features are no longer correlated. The correlation length scales as ξ ~ *t*^1*/z*^ where the dynamic exponent is defined as *z* = *α/β.*

Although the roughening process during growth of thin films is microscopically diverse and complex in nature, a number of studies on growth of thin films have revealed that the interface roughness follows the Family-Vicsek scaling ansatz. However, in recent years, many experimental and theoretical studies have reported scaling patterns very different from that predicted by the Eq. (2)^11^,^12^,^13^,^14^,^15^,^16^,^17^,^18^,^19^,^20^,^21^,^22^. In such cases, growth models with different exponents at long (global/saturated) and short (local) length scales have been suggested. While the global width *w*(*L,t*) still follows the Family-Vicsek scaling relation (Eq. (2)), the local width is represented by the *anomalous scaling* ansatz:
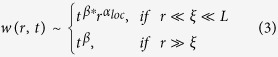
where *α*_*loc*_ is the local roughness exponent and β* is the anomalous growth exponent indicating the time dependence of the local width at length scales smaller than *ξ* and is given by *β* − *β*_*loc*_ = (*α* − *α*_*loc*_)*/z*. The time dependence of *w*(*r, t*) through the term 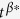 is the crucial signature of the anomaly in the scaling behaviour.

Although extensive studies have been carried out, the understanding of the surface dynamics of thin films is far from complete, as evident from the wide scatter in the experimentally determined scaling exponents for a given technique^4^. In particular, growth mechanisms in films by chemical bath deposition (CBD) technique on the lines of the dynamic scaling theory has not been explored, in spite of the fact that the CBD has been established as one of the most preferred techniques for cost effective large area deposition of semiconductor thin films^23^. This work is the first ever study of dynamics of surface evolution in compound semiconductor thin films grown by the CBD film.

We have investigated roughening of CdS thin films which were grown from an ammonia free chemical bath. Over the years, CdS thin films have emerged as a binary semiconductor of high technological importance and have found applications in photovoltaics, lasers, non-linear optical devices, etc^24^,^25^,^26^. Using the kinetic roughening analysis, we show an anomalous scaling pattern of the growth front of the chemical bath deposited CdS films. It is expected that this work will provide important clues for the complex changes of kinetic roughening that occur during the formation of CBD thin films.

## Results and Discussion

**Figure 1:**
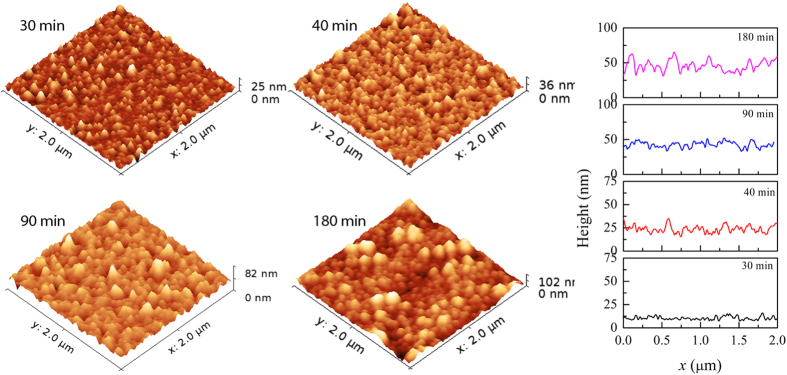
2 μm × 2 μm AFM images of CBD CdS thin films grown under identical experimental conditions for different durations and the corresponding line profiles across the scan areas.

Figure 1 shows typical surface morphology of the films of increasing deposition times. A granular structure with even sized grains, typical of polycrystalline thin films is observed at all stages of film growth. From the images, both surface roughening and coarsening process are evident, which is supported from the time sequences of scanned profile of the surfaces wherein the vertical and lateral stretch of the particles increased with the increase in deposition time.

**Figure 2:**
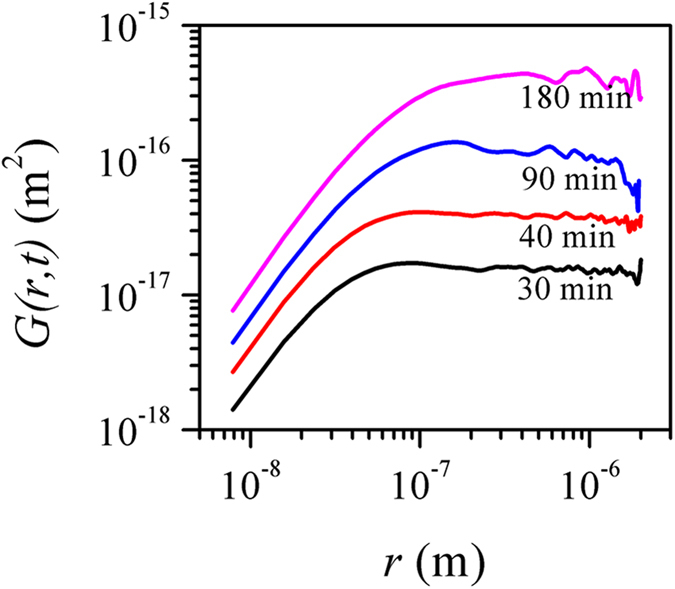
Log-log plot of the height-height correlation function G(*r*, *t*) estimated for the films of different deposition times.

**Figure 3:**
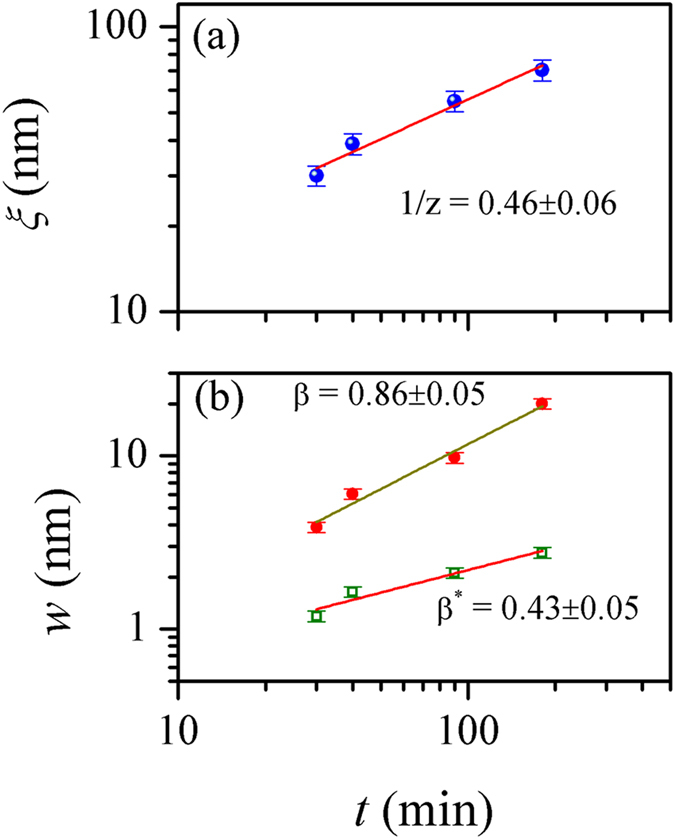
Logarithmic plots of (**a**) lateral correlation length *ξ* and (**b**) interface width *w* versus deposition time. In the bottom panel, solid circles (**⚫**) and open squares (□) represent the data points corresponding to global width and the local width, respectively. Solid lines in both panels are the linear fits to the data. The values of the slopes are indicated.

The growth dynamics and whether roughening of the growth front follows any scaling pattern were assessed from the height-height correlation function *G*(*r*, *t*) defined as 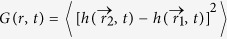 for each film, which are presented in Fig. 2. *G*(*r*, *t*) shows a power law dependence on *r* for small length scales and remains saturated at large *r* values for films of varying deposition times. More importantly, the curves were up-shifted as deposition time increased at all length scales. This, however, is in contrast with the systems following the Family-Vicsek relation, wherein *G*(*r*, *t*) ~ [*w*(*r*, *t*)]^2^ exhibit behaviors typical of self-affine interfaces: *G*(*r*, *t*) scales as *G*(*r*, *t*) ~ *r*^2*α*^ for *r* ≪ *ξ* and becomes constant for *r* ≫ *ξ*. The up-lifting of the curves at small length scales indicates the time dependence of *G*(*r*, *t*) and hence, the anomaly in the scaling pattern. Consequently, *G*(*r*, *t*) is expected to follow the scaling relation as given in Eq. (3), i.e.,
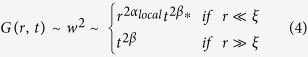


The dynamic exponent *z* was estimated from the time dependent variation of the correlation length *ξ,* which was accurately determined from the relation Γ(*r* = *ξ*)*/*Γ(*r* = 0) = e^−1^ after computing the auto-correlation function Γ(*r*, *t*) from the AFM images corresponding to different deposition times (not shown here)^27^. The plot of the variation of *ξ* with deposition *t* (Fig. 3(a)) reveals the scaling of *ξ* as *ξ* ~ *t*^*1/z*^ with 1/*z* = 0.46 ± 0.06. The global and local surface widths were estimated from the height-height correlation functions as per Eq. (4) and are shown as function of deposition time in Fig. 3(b). Both global and local surface widths show a power-law dependence on growth time, typical of anomalous dynamic scaling patterns. The least-square linear fits to the data points yield that the global surface width grew with *β* = 0.86 ± 0.05, much faster than the random deposition limit of stochastic roughening (*β* = 0.5). The local surface width, on the other hand increased less rapidly at *β*_*loc*_ = *β* − β* = 0.43 ± 0.10. The fact β* ≠ 0 confirms the anomalous scaling pattern in the system. The global roughness exponent *α* = *βz* is calculated to be 1.87 ± 0.35. The observed rapid roughening of the CBD film surface characterized by a high *β* value is similar to the cases of films grown by sputtering^22^.

**Figure 4:**
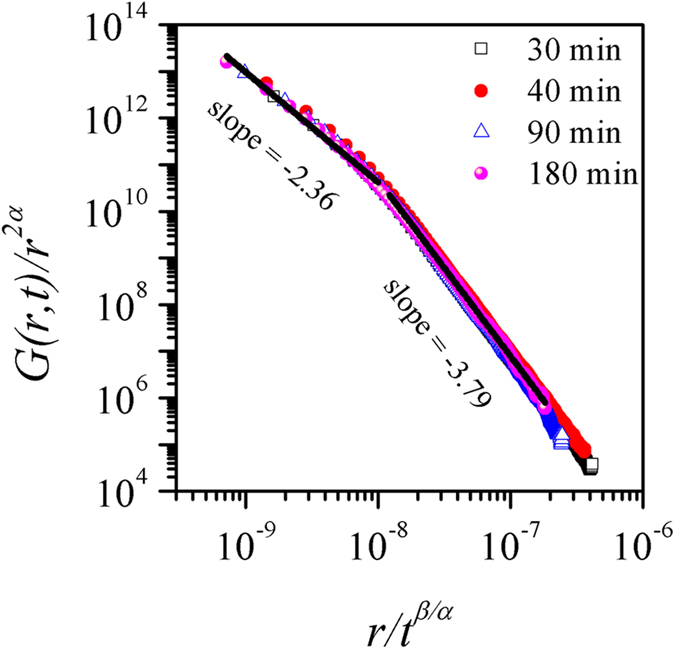
Logarithmic plot of *G*(*r*, *t*)/r^2α^ versus r/t^β/α^ for different growth times showing a good data collapse of the height-height correlation function. The solid lines are linear fits to the data points.

The consistency of our analysis was verified by collapsing the height-height correlation functions obtained for films of different growth times. As Eq. (4) indicates, the plot of curves *G*(*r*, *t*)/*r*^*2α*^ versus *r*/*t*^*β/α*^ for all growth times should collapse, with the slopes in small and high arguments being equal to m_1_ = −2(*α* − *α*_*loc*_) and m_2_ = −2*α*, respectively. We show in Fig. 4 that all data points collapse into a single curve confirming the integrity of the general dynamic scaling theory as given in Eq. (4). The slope of the curve in small and high arguments was found to be m_1_ = −2.36 and m_2_ = −3.79, respectively. It is noted that m_1_ ≠ 0 implies *α* *≠* *α*_*loc*_, and therefore, the anomalous scaling behavior in our system. Using the values of m_1_ and *α*, *α*_*loc*_ was calculated to be 0.69, which is close to the value 0.78 ± 0.07 calculated from the slope of the *G*(*r*, *t*) vs *r* curves (Fig. 2) for *r* ≪ *ξ* as per Eq. (4).

**Figure 5:**
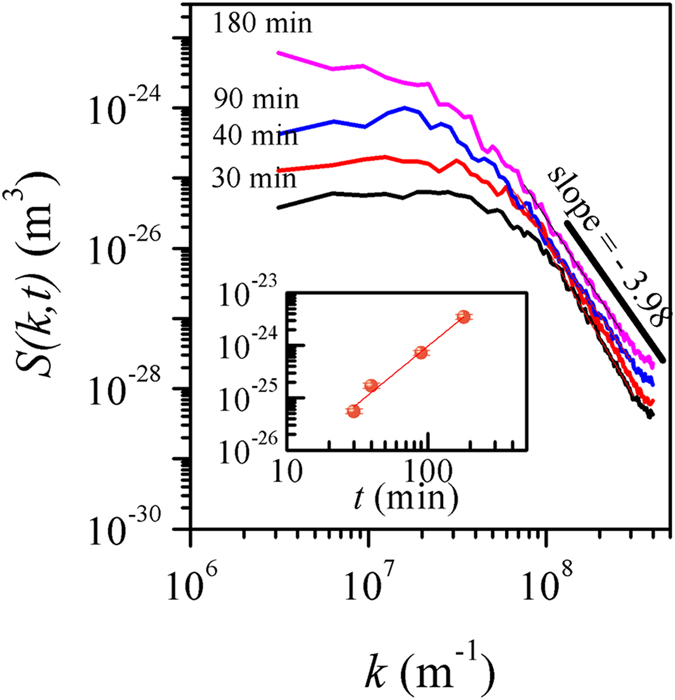
Logarithmic plot of power spectral density function S(*k*, *t*) versus wave number *k* for different growth times. The slope of the curves for large *k* values is indicated. Inset: Time dependence curve of *S*(*k*, *t*) in the low *k*-regime. The line denotes the linear fit to the data points.

More insight into the dynamics of the roughening process in our system was gained from the plot of the power spectral density (PSD) functions for films of different deposition times, as shown in Fig. 5. All PSD curves show two distinct regimes: the power law *k* dependence in the high-*k* regime crosses over to a *k*-independent regime at *k*_*c*_ ~ 1/*ξ* as the surface features lose their correlation. The dynamic scaling behavior in a (2+1)-dimension system is manifested in the PSD as *S*(*k*, *t*) ~ *t*^(*2α+2*)*/z*^ in the low *k*-regime and as *S*(*k*, *t*) ~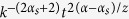 in the high *k*-regime for *k* ≫ *k*_*c*_ where *α*_*s*_ is the spectral roughness exponent^15^. As seen from the figure, the vertical shift of the PSD curves, especially for high *k-*regime, as the deposition time increased suggests that *α* ≠ *α*_*s*_. The average value of the spectral roughness exponent was obtained as *α*_*s*_ = 1.49 ± 0.22 by measuring the slope of the *k*-dependent PSD plot in the high-*k* regime. The global rougheness exponent *α* can be determined from the time dependence of *S*(*k*, *t*) in the low *k*-regime, which is shown as inset to Fig. 4. From the slope, *α* is found to be 2.20 ± 0.08 which is close to the value independently determined from the scaling relation *α* = *βz*, as discussed above, using the height-height correlation and autocorrelation functions.

The major objectives of the studies of scaling patterns include identification of universality classes and the roughness controlling mechanisms in the growth of thin films based on the evaluated scaling exponents. However, it has not been always straightforward to assign a universality class for films grown in a specific technique. In our case for the CBD CdS thin films, we find that the dynamic evolution of roughness follows a complex scaling behaviour characterized by exponents *α*_*loc*_ = 0.78 ± 0.07, *α* = 2.20 ± 0.08, *α*_*s*_ = 1.49 ± 0.22, 1/*z* = 0.46 ± 0.06, *β* = 0.86 ± 0.05 and *β*_*loc*_ = 0.43 ± 0.10. The scaling relationship α_loc_ ≠ α ≠ α_*s*_ does not belong to any of the known classes of interfacial growth^1^,^13^,^15^ and indicates a new class^15^.

Although the exact mechanism has not been well understood, some studies in recent years have proposed that the anomalous kinetic roughening of the surfaces with a high growth exponent arises as consequences of nonlocal effects^28^. Nevertheless, a clear universality class could not be defined yet. In the case of sputter-deposited thin films, shadowing arising due to the angular spread of the impinging particles works as a nonlocal effect, which led to rapid roughening of the surfaces (i.e., with a high *β* value)^16^,^22^,^29^. The shadowing effect, however, cannot be a possible explanation in the present study on CBD thin films.

In the case of solution based methods, which are technologically more attractive, the growth of thin films is inherently complex than the vacuum-based ones, for example MBE. In the few studies on films grown by electroless and electrodeposition techniques, the obtained values of the exponents indicate significant deviation from the universality class as predicted from the KPZ equation^30^,^31^,^32^,^33^,^34^. The exponents obtained in this work are similar to those found in refs 30, 31, 32, 33, 34, which suggests that a similar mechanism may govern the dynamic evolution of the roughness of the films studied here. In other words, these exponents might be associated with nonlocal bulk diffusion effects^30^,^31^,^32^,^33^,^34^,^35^.

## Conclusion

Kinetic roughening of CdS thin films grown by the technologically viable chemical bath deposition technique has been studied using the generic scaling *ansatz*. The analyses revealed anomalous scaling of roughness with rapid roughening of the surfaces as a consequence of bulk diffusion instability. The characteristic exponents *α*_*loc*_ = 0.78 ± 0.07, *α* = 2.20 ± 0.08, *α*_*s*_ = 1.49 ± 0.22, 1/*z* = 0.46 ± 0.06, *β* = 0.86 ± 0.05 and *β*_*loc*_ = 0.43 ± 0.10 as determined using the real and Fourier space correlation functions cannot be related to any known universality classes based on local growth models, suggesting that nonlocal effect plays an important role in the evolution of growth front of the chemical bath deposited thin films. In the light of previous studies on electroless and electrodeposited films, the present results indicate that nonlocal effects in the form of diffusional instability should be incporporated in the models to further our understanding of growth mechanism of the films.

## Methods

The precursor solution was prepared by sequential addition of 25 ml of 0.1 M cadmium chloride (Loba Chemie, India, 99.0%), 20 ml of 1 M sodium citrate (Loba Chemie, India, 98.0%), 5 ml of 1 M potassium hydroxide (Loba Chemie, India, 85.0%) and 20 ml of buffer (NaOH) pH = 10 (Loba Chemie, India, 98.0%) in a beaker. The solution was added with DI water to complete a total volume of 90 ml. The bath temperature was increased from room temperature and after reaching a bath temperature of 40 °C, 10 ml of 1 M thiourea (Loba Chemie, India, 99.0%) was added. The deposition temperature was maintained at 40 °C. The glass substrate was kept vertical in the beaker. A homogeneous layer with dark yellow color and with good adhesion to substrate of thicknesses depending on deposition time was obtained. After the deposition, the films were taken out of the bath and washed in DI water ultrasonically to remove the loosely adhered particles on the film and finally dried in air.

The surface topography of the films were studied in detail by atomic force microscopy (AFM) using a NT-MDT-NTEGRA PRIMA system. The images were acquired in air at room temperature in tapping mode of operation and at 256 × 256 pixels. Images were recorded at least at four different points on the sample surfaces and the results were consistent.

## Additional Information

**How to cite this article**: Gupta, I. and Mohanty, B. C. Dynamics of surface evolution in semiconductor thin films grown from a chemical bath. *Sci. Rep.* **6**, 33136; doi: 10.1038/srep33136 (2016).

## References

[b1] Barabási L. & Stanley H. E. Fractal Concepts in Surface Growth (Cambridge University Press, 1995).

[b2] Meakin P. Fractals, Scaling and Growth Far From Equilibrium (Cambridge University Press, 1998).

[b3] Krim J. & Palasantzas G. Experimental observations of self-affine scaling and kinetic roughening at sub-micron length scales. Int. J. Mod. Phys. B 9, 599–632 (1995).

[b4] Munoz M. A. Multiplicative noise in non-equilibrium phase transitions: A tutorial In Advances in Condensed Matter and Statistical Physics , (eds Korutcheva E. & Cuerno R. ) 37–68 (Nova Science, 2004).

[b5] Steudel S. . Influence of the dielectric roughness on the performance of pentacene transistors. App. Phys. Lett. 85, 4400–4402 (2004).

[b6] Rosengren A., Bjursten L. M., Danielsen N., Persson H. & Kober M. Tissue reactions to polyethylene implants with different surface topography. J. Mater. Sci.: Mater. Med. 10, 75–82 (1999). 15347928 10.1023/a:1008964819101

[b7] Berginski M. . The effect of front ZnO:Al surface texture and optical transparency on efficient light trapping in silicon thin-film solar cells. J. Appl. Phys. 101, 074903 (2007).

[b8] Gao H. & Nix W. D. Surface roughening of heteroepitaxial thin films. Annu. Rev. Mater. Sci. 29, 173–209 (1999).

[b9] Kardar M., Parisi G. & Zhang Y. C. Dynamic scaling of growing interfaces. Phys. Rev. Lett. 56, 889–892 (1986). 10033312 10.1103/PhysRevLett.56.889

[b10] Family F. & Vicsek T. Dynamics of Fractal Surfaces (World Scientific, 1991).

[b11] Va´zquez J., Salvarezza R. C. & Arvia A. J. Validity of the linear growth equation for interface evolution for copper electrodeposition in the presence of organic additives. Phys. Rev. Lett. 79, 709–712 (1997).

[b12] Roberds B. E. & Farrens S. N. An atomic force microscopy study on the roughness of silicon wafers correlated with direct wafer bonding. J. Electrochem. Soc. 143, 2365–2371 (1996).

[b13] Lo´pez J. M., Rodrı´guez M. A. & Cuerno R. Superroughening versus intrinsic anomalous scaling of surfaces. Phys. Rev. E 56, 3993–3998 (1997).

[b14] Castro M., Cuerno R., Sa´nchez A. & D-Adame F. Anomalous scaling in a nonlocal growth model in the Kardar-Parisi-Zhang universality class. Phys. Rev. E 57, 2491(R)–2494(R) (1998).

[b15] Ramasco J. J., Lo´pez J. M. & Rodrı´guez M. A. Generic dynamic scaling in kinetic roughening. *Phys. Rev. Lett*. 84, 2199–2202 (2000). 11017243 10.1103/PhysRevLett.84.2199

[b16] Pand N. & Tzeng W. Interfaces with superroughness. Phys. Rev. E 61, 3559–3563 (2000). 10.1103/physreve.61.3559 11088132

[b17] Lo´pez J. M. & Scmittbuhl J. Anomalous scaling of fracture surfaces. Phys. Rev. E 57, 6405–6408 (1998).

[b18] Morel S., Schmittbuhl J., Lo´pez J. M. & Valentin G. Anomalous roughening of wood fractured surfaces. Phys. Rev. E 58, 6999–7005 (1998).

[b19] Yang N. H., Wang G. C. & Lu T. M. Instability in low-temperature molecular-beam epitaxy growth of Si/Si(111). *Phys. Rev. Lett*. 73, 2348–2351 (1994). 10057037 10.1103/PhysRevLett.73.2348

[b20] Liu Z.-J., Jiang N., Shen Y. G. & Mai Y.-W. Atomic force microscopy study of surface roughening of sputter-deposited TiN thin films. *J. Appl. Phys*. 92, 3559–3563 (2002).

[b21] Yang J. J., Liu B., Wang Y. & Xu K. W. Homologous temperature dependence of global surface scaling behaviors of polycrystalline copper films. *Appl. Phys. Lett*. 95, 194104 (2009).

[b22] Auger M. A. . Intrinsic anomalous surface roughening of TiN films deposited by reactive sputtering. Phys. Rev. B 73, 045436 (2006).

[b23] Hodes G. Chemical Solution Deposition of Semiconductor Films . (Marcel Dekker, 2002).

[b24] Brus L. E. Quantum crystallites and nonlinear optics. Appl. Phys. A 53, 465–474 (1991).

[b25] Kale R. B., Sartale S. D., Chougule B. K. & Lokhande C. D. Growth and characterization of nanocrystalline CdSe thin films deposited by the successive ionic layer adsorption and reaction method. Semicond. Sci. Technol. 19, 980–986 (2004).

[b26] Rossetti R., Hill R., Gibson J. M. & Brus L. E. Excited electronic states and optical spectra of ZnS and CdS crystallites in the ≊15 to 50 Å size range: Evolution from molecular to bulk semiconducting properties. J. Chem. Phys. 82, 552–559 (1985).

[b27] Siniscalco D., Edely M., Bardeau J. F. & Delorme N. Statistical Analysis of mounded surfaces: Application to the evolution of ultrathin gold film morphology with deposition temperature. Langmuir 29, 717–726 (2013). 23252843 10.1021/la304621k

[b28] López J. M., Castro M. & Gallego R. Scaling of local slopes, conservation laws, and anomalous roughening in surface growth. Phys. Rev. Lett. 94, 166103 (2005). 15904249 10.1103/PhysRevLett.94.166103

[b29] Mohanty B. C., Choi H. R. & Cho Y. S. Fluctuations in global surface scaling behavior in sputter-deposited ZnO thin films. Euro. Phys. Lett. 93, 26003 (2011).

[b30] Huo S. & Schwarzacher W. Anomalous scaling of the surface width during Cu electrodeposition. *Phys. Rev. Lett*. 86, 256–259 (2001). 11177805 10.1103/PhysRevLett.86.256

[b31] Aogaki R., Kitazawa K., Kose Y. & Fueki K. Theory of powdered crystal formation in electrocrystallization - occurrence of morphological instability at the electrode surface. Electrochem Acta 25, 965–972 (1980).

[b32] Xu Y. . Anomalous scaling in amorphous PdNiP films elecrodeposition. *J. Electrochem. Soc*. 155, D731–D733 (2008).

[b33] Saitou M. Anomalous scaling of nickel surfaces in pulse-current electrodeposition growth. Phys. Rev. B 66, 073416 (2002).

[b34] Hasan N. M., Mallett J. J., dos Santos Filho S. G., Pasa A. A. & Schwarzacher W. Dynamic scaling of the surface roughness of Cu deposited using a chemical bath. Phys. Rev. B 67, 081401 (2003).

[b35] Lee I. J., Yun M., Lee S. M. & Kim J. Y. Growth mechanism of vapor born polymer films. Phys. Rev. B 78, 115427 (2008).

